# Is there a correlation between levels of incapability and athletic identity?

**DOI:** 10.1371/journal.pone.0359550

**Published:** 2026-09-30

**Authors:** Alexander Drost, Jakob Bleacher, David Ring, Amin Razi, Jefferson Hunter, Aydin Azarpey

**Affiliations:** Department of Surgery and Perioperative Care, The University of Texas at Austin, Austin, Texas, United States of America; Adnan Menderes Universitesi, TÜRKIYE

## Abstract

**Objectives:**

Among people seeking sports-specific musculoskeletal specialty care we sought to determine 1) whether level of athletic identity mediates or moderates the relationship between thoughts and feelings and levels of capability and pain intensity, and 2) whether levels of pain intensity and capability differ among statistical groupings based on age and mindsets including athletic identity.

**Methods:**

Adult patients seeing a sports medicine specialist (n = 135) completed measures of levels of capability, pain intensity, thoughts and feelings regarding sensation, and identification as an athlete. We planned mediation/moderation analysis. When that was not feasible, weadded an unplanned, exploratory cluster analysis of mindset factors.

**Results:**

The lack of correlation between athletic identity and levels of capability and pain intensity precluded moderation/mediation analysis. An exploratory cluster analysis suggested that relatively younger people who identify as athletes and middle-aged people with somewhat greater feelings of distress may have greater pain intensity on average.

**Conclusions:**

The finding that feelings of distress, independent of athletic identity, are associated with greater pain intensity and incapability is consistent with evidence that mindsets are a key aspect of musculoskeletal health, perhaps independent of specific identities.

## Introduction

### Background

Narrative identity is the internal and evolving autobiographical story of oneself used to construct a perception of the past and anticipate one’s future [1]. Interpretation of the body’s changes and sensations occurs in the context of one’s narrative identity. Levels of interpretation of sensations as a threat to one’s narrative identity (unhelpful thoughts or less healthy mindsets) account for notable amounts of the variation in levels of capability and comfort, and more so than levels of pathophysiology severity [2–6]. For instance, the body may be interpreted as damaged or vulnerable. But the mind may not be aware that this is a theory or interpretation and instead may consider these thoughts facts (cognitive fusion) and a representation of reality. Instead, a person may only notice the change in narrative identity. For instance, rather than “it hurts to do ___, and I’m not sure it’s safe to do it,” the narrative is “I can no longer be the person who does ____.”

### Rationale

People who identify as athletes may regard musculoskeletal discomfort as incompatible with athletics. Such an association might limit their capability. Musculoskeletal sensations are often from age-related changes (senescence), idiopathic pathophysiology, residual impairment from prior damage, or part of the normal symptom trajectory during recovery from damage. Interpretation of sensations from these conditions as an indication of harm or vulnerability—in other words, that symptomatic activities are unhealthy and are off limits—is a form of unhelpful or distorted thinking (unhealthy misconceptions) that is notably associated with greater symptom intensity [5]. For instance, as a person develops arthritis and rotator cuff tendinopathy with age, they may limit their athletic activity if they misinterpret the harmless sensations from these degenerative, senescent pathophysiologies as indicating that painful athletic activities are off limits. They may not notice the misinterpretation. They may only experience the loss of the cherished role (athletic identity). They may seek care motivated by the false belief that they need to eliminate the sensations in order to maintain their athlete identity. In most cases, this is an incorrect or unhelpful thought, and an accurate interpretation of the sensations and an evolution of one’s identity towards “aging athlete” is sufficient to maintain one’s athlete identity. The process of evolving one’s narrative identity to match one’s body is particularly important when the pathophysiology is irreversible as is nearly always the case for senescent pathophysiologies. Medical care has the potential to harm health by reinforcing these common misconceptions, for instance, by labeling degenerative changes in the knee meniscus or rotator cuff as “tears” [7,8].

Clinicians can be attuned to aspects of illness that can be characterized as a form of discordance between a person’s ideal and actual self. In other words, the musculoskeletal symptoms may be less a target for biomedical treatments, and more so the nidus of an identity crisis. Awareness and anticipation of these aspects of illness could redefine health strategies away from a central focus on biomedical treatments intended to alleviate or resolve symptoms. We might continue to strategize more inclusive, biopsychosocial efforts intended to help people return to their cherished roles in part by cultivating a healthier inner narrative about their body and its sensations. This study explores the possibility that unhelpful thoughts and feelings of distress regarding sensations might have a stronger relationship to higher levels of discomfort and incapability based on the degree to which people align with specific inner narratives such as identifying as an athlete. In other words, the degree to which a person identifies as an athlete might increase the correlation between misinterpretations (unhelpful thoughts) regarding sensations and what a person feels they can or should do.

### Questions

In a cross-sectional study of people presenting to a musculoskeletal sports specialist we asked: 1) Does level of athletic identity mediate or moderate the relationship between thoughts and feelings and level of capability and pain intensity? And in an unplanned, exploratory analysis, 2) What is the association between levels of pain intensity and capability and statistical clusters based on age, mindset (distress, unhelpful thinking), and athletic identity among people seeking musculoskeletal specialty care?

## Materials and methods

### Study design and settings

New and returning patients seeking musculoskeletal specialty care from a unit or surgeon marketed as providing “sports medicine” care in an urban region of the United States who spoke either English or Spanish were invited to participate in this cross-sectional study between August and November 2024. The study was reviewed and approved by the University of Texas at Austin Institutional Review Board. Few patients declined, and declines were not tracked. Verbal informed consent was obtained from each participant by a member of the research team prior to survey completion. The University of Texas at Austin Institutional Review Board approved the use of verbal consent, combined with completion of the questionnaires representing informed consent, and formal written consent was not required. A researcher then asked the participant to complete a survey on the encrypted REDCap (Vanderbilt University; Nashville, TN) platform. Patients were excluded if they had any cognitive or physical difficulty precluding completion of the survey on a tablet.

All participants were asked to complete measures of athletic identity, pain intensity, unhelpful thoughts regarding sensations, feelings of distress regarding sensations, levels of capability, and demographics. The researcher entered the diagnosis, anatomical region, pathophysiology category, and language.

### Participants

Of the 135 participants 68 (50%) were men and the mean age was 42 years (Table 1).

**Table 1:** Demographics of 135 participants.

Variable	% (N)
	
**Gender**	
Women	48% (65)
Men	50% (68)
Another gender identity	1% (1)
Prefer not to answer	1% (1)
**Office**	
1	83% (112)
2	17% (23)
**Pathophysiology**	
Traumatic	49% (66)
Non-traumatic	51% (69)
**Language**	
English	81% (111)
Spanish	19% (24)
**Marital Status**	
Married	41% (55)
Divorced	7.4% (10)
Single (never married)	46% (62)
Separated	3% (4)
Widowed	1.5% (2)
Prefer not to answer	1.5% (2)
**Education**	
Elementary school	7% (9)
High school	31% (42)
2-year college	11% (15)
4-year college	27% (37)
Post-college graduate degree	20% (27)
Prefer not to answer	4% (5)
**Work status**	
Employed	57% (77)
Unemployed	17% (23)
Disabled	6% (8)
Prefer not to answer	4% (5)
Other (e.g., student, homeworker, or retired)	16% (22)
**Income**	
Less than $15,000	12% (16)
From $15,000 to $29,999	11% (15)
From $30,000 to $49,999	10% (13)
From $50,000 to $99,999	15% (20)
More than 100.000	22% (30)
Prefer not to answer	30% (41)
**Insurance**	
Medicaid	2% (3)
Self-pay (no health insurance)	3% (4)
Private	35% (47)
Medicare	11% (15)
County safety-net	37% (50)
Other	6% (8)
Prefer not to answer	6% (8)
**Anatomical location**	
Upper extremity	34% (47)
Lower extremity	63% (84)
Spine	3% (4)
**Do you consider yourself an athlete?**	
Yes	54% (72)
No	46% (63)
	
**Variable**	**Mean ± SD**
**Age**	42 ± 16
**Capability (PROMIS** **PF** **CAT**)	41 ± 8.6
**Athletic Identity Measurement Scale**	24 ± 11
**Unhelpful Thoughts**	7.8 ± 2.8
**Feelings of Distress**	7.5 ± 3.5
**Pain Intensity**	5.2 ± 2.7

SD = standard deviation; PROMIS PF CAT = Patient Reported Outcome Measurement Information System’s Physical Function Computer Adaptive Testing is a measure of capability with a mean score of 50 representing the United States population norm with every 10 points above or below 50 representing a standard deviation difference better or worse than the population mean respectively; The Athletic Identity Measurement Scale is that measures to which degree a person identifies as an athlete that ranges from 7 to 49, with greater scores representing stronger identity as an athlete. A mean score of 24 represents a moderate level of identification as an athlete; We used 3 items each to measure about unhelpful thoughts and feelings of distress regarding sensations resulting in scores that range from 3 to 15 with higher scores representing less healthy thoughts and feelings. Mean scores of 7.8 and 7.5 indicate moderate unhelpful thinking and feelings of distress.

### Response variables

Level of capability was measured using the Patient-Reported Outcomes Measurement Information System (PROMIS) Physical Function Computer Adaptive Test (PF CAT). On computer adaptive tests, each response determines the next question, and as few as 4 or 5 responses can lead to a final score. The score is scaled based on the general American population with a score of 50 representing the population mean and every 10 points higher or lower representing a standard deviation. We measured pain intensity using an 11-point ordinal scale from 0, representing no pain, to 10, representing the worst imaginable pain.

### Explanatory variables

The Athletic Identity Measurement Scale (AIMS) measures the degree to which a person identifies as an athlete. Unhelpful thoughts and feelings of distress were each measured using three validated items identified in a factor analysis and rated on a 5-point Likert scale from 1, strongly disagree, to 5, strongly agree [5]. The resulting scores ranged from 3 to 15 with higher scores representing greater unhelpful thoughts or greater distress regarding sensations. The demographic variables recorded were age, gender, language, insurance type, marital status, education level, household income, and identification as an athlete (yes or no). The musculoskeletal condition was categorized as traumatic or nontraumatic and the anatomical region as upper extremity, lower extremity, or spine.

### Statistical analysis

We performed descriptive statistics of all variables. Normal continuous variables were described in terms of means and standard deviations. Percentages with numbers were used for categorical variables. For categorical variables such as income, “Prefer not to answer” was an available response option and was analyzed as a distinct category rather than treated as missing data.

For bivariate analysis, we used a Student T-test for dichotomous variables and one-way analysis of variance (ANOVA) for other categorical variables. Linear regression was performed for continuous response variables. Analysis was performed using a combination of the Excel Data Analysis Package and Stata (Version 14.2, StataCorp, College Station, Texas). Groups with fewer than 20 observations were pooled for the purpose of analysis.

We planned to analyze the mediation and moderation effects of athletic identity on the relationship between thoughts and feelings regarding sensations with level of capability and pain intensity. We did not perform a multivariable analysis. The variables that would enter such a model are related, raising concern for collinearity that can produce unstable estimates, and athletic identity had no bivariate association with either pain intensity or capability, so it could not contribute meaningfully to a multivariable model.

We also performed an exploratory cluster analysis (k-means) to identify groups of patients with comparable levels of 1) feelings of distress about symptoms, 2) unhelpful thoughts about symptoms, 3) athletic identity, and 4) age. We allowed up to 20 clusters to be generated and assessed scree plots to identify the optimal number of clusters to retain using the “elbow” method [9]. This approach allowed us to assess the mean differences in a) pain intensity, and b) level of incapability among groupings of patients with similar mental health using one-way analysis of variance (ANOVA) to assess the mean differences in the outcomes among the identified groupings of mental health.

An a priori sample size calculation indicated that 130 patients would provide 80% statistical power based on linear regression with ten variables, with alpha set at 0.05, if the outcome variables accounted for 5% or more of the AIMS score and if the complete model accounted for 15% or more of the overall variability. We enrolled an additional 10 patients to account for potential incomplete responses, for a total sample size of 140 patients. Due to an oversight, this calculation was erroneously framed around multivariable linear regression rather than moderation analysis, leaving the study underpowered for the latter. As an additional check, assuming a medium effect size (Cohen’s f^2^ = 0.15), a two-sided significance level of 0.05, 80% statistical power, and eight predictor variables, the required sample size was estimated at 109 participants, which our sample met. However, because athletic identity was not associated with pain intensity or capability in bivariate analysis, the mediation/moderation analysis was not conducted, and this oversight had no bearing on our results or conclusions.

## Results

### Does level of athletic identity mediate or moderate the relationship between thoughts and feelings and level of capability and pain intensity?

There was no correlation between level of athletic identity measured on a continuum or dichotomized athletic identity and level of capability and pain intensity, so mediation and moderation analyses were not possible (Table 2). There were notable correlations between levels of capability and levels of unhelpful thoughts regarding sensations (r = −0.42, *P* < 0.001) and feelings of distress regarding sensations (r = −0.59, *P* < 0.001), and also notable correlations between pain intensity and unhelpful thoughts regarding sensations (r = 0.37, *P* < 0.001) and feelings of distress regarding sensations (r = 0.63, *P* < 0.001).

**Table 2:** Bivariate analysis of factors associated with levels of discomfort (pain intensity) and capability.

	Capability		Pain intensity	
Variable	Pearson Correlation	P-value	Pearson Correlation	P-value
**Age**	−0.11	0.20	−0.04	0.67
**Identification as athlete**	0.12	0.16	−0.04	0.67
**Unhelpful thoughts regarding sensations**	−0.42	**<0.001**	0.37	**<0.001**
**Distress regarding sensation**	−0.59	**<0.001**	0.63	**<0.001**
	**Mean ± SD**	**P-value**	**Mean ± SD**	**P-value**
**Gender**		0.36		0.13
Women	42 ± 8.1		5.5 ± 2.8	
Men	41 ± 9.4		4.8 ± 2.5	
**Clinic**		0.61		0.61
1	41 ± 9.2		5.1 ± 2.7	
2	42 ± 6.2		5.4 ± 2.7	
**Pathophysiology**		0.54		0.08
Traumatic	41 ± 8.7		4.7 ± 2.8	
Non-traumatic	42 ± 8.6		5.5 ± 2.5	
**Language**		0.06		**0.003**
English	42 ± 8.8		4.8 ± 2.6	
Spanish	38 ± 8.1		6.6 ± 2.7	
**Marital Status**		0.51		1.0
Married	41 ± 8.7		5.1 ± 2.9	
Other	40 ± 10		5.2 ± 2.3	
Single (never married)	41 ± 8.7		5.1 ± 2.5	
**Education**		0.10		**0.006**
High school	40 ± 8.0		5.8 ± 2.5	
4-year college	44 ± 10		4.4 ± 2.9	
Post-college graduate degree	43 ± 7.8		4.8 ± 2.3	
Prefer not to answer	39 ± 7.7		5.5 ± 2.9	
**Work status**		**0.02**		**0.02**
Employed	43 ± 8.8		4.9 ± 2.5	
Unemployed	38 ± 7.8		6.5 ± 2.2	
Other (student, homemaker, retired)	40 ± 8.5		4.8 ± 3.0	
**Income**		0.21		0.26
Less than 30,000	39 ± 9.1		5.6 ± 3.2	
From 30,000–99,999	43 ± 7.9		4.5 ± 2.6	
More than 100,000	44 ± 8.8		4.6 ± 2.2	
Prefer not to answer	40 ± 8.8		5.7 ± 2.6	
**Insurance**		0.14		0.13
Medicare	39 ± 7.4		4.4 ± 3.0	
Private	44 ± 9.5		4.4 ± 2.5	
County safety net	39 ± 8.4		6.0 ± 2.5	
Other	42 ± 7.9		5.1 ± 2.7	
**Condition location**		**0.04**		0.06
Upper extremity	39 ± 7.2		5.7 ± 2.4	
Lower extremity	42 ± 9.3		4.9 ± 2.8	
**Do you consider yourself an athlete?**		0.42		0.93
Yes	42 ± 9.1		5.0 ± 2.7	
No	41 ± 8.5		5.2 ± 2.7	

Bold = p < 0.05; Capability measured with PROMIS Physical Function Computer Adaptive Test; Pain intensity measured using an 11-point ordinal scale. Factors associated with higher levels of discomfort and incapability include distress regarding sensations (large correlations), unhelpful thinking regarding sensations (medium correlations), and social disadvantage (safety net insurance, Spanish speakers, unemployed—all medium to large effect sizes [ratio of difference in means over standard deviation]).

### Relationship of statistical groupings of age and mindset to athletic identity

An exploratory cluster analysis identified four statistical groups of participants on the basis of similar means for age, athletic identity, unhelpful thoughts, and feelings of distress (Table 3). Group 1 had a mean age of 32 and a relatively strong athletic identity (younger adults with stronger athletic identity), but average scores of feelings of distress and unhelpful thoughts. Group 2 was relatively young (mean age, 24), had lower feelings of distress on average, average athletic identity, and average unhelpful thoughts (younger adults with lower feelings of distress). Group 3 had a mean age of 62, lower athletic identity and somewhat greater unhelpful thoughts on average (olderadults with lower athletic identity and somewhat greater unhelpful thoughts). And Group 4 was middle-aged (mean age 44), with lower athletic identity, greater feelings of distress, and lesser unhelpful thinking on average (middle-aged adults with lower athletic identity and greater feelings of distress).

**Table 3:** Association of levels of pain intensity and capability with statistical groupings of relative athletic identity, feelings of distress, unhelpful thoughts, and age.

	Tendency to identify as an athlete	Feelings of distress regarding sensations	Unhelpful thoughts regarding sensations	Age	Pain Intensity	Capability
					Mean ± SD	
					p = 0.02	p = 0.25
**Group 1**	z = 1.3	z = −0.04	z = −0.01	z = −0.7	5.2 (2.7)	43 (10)
n = 36	Mean = 39 (5.7)	Mean = 7.5 (3.7)	Mean = 7.7 (2.6)	Mean = 32 (8.4)		
(27%)						
**Group 2**	z = 0.03	z = −0.4	z = −0.05	z = −1.2	4.0 (2.4)	43 (6.6)
n = 22	Mean = 25 (5.6)	Mean = 6.3 (2.8)	Mean = 7.6 (2.7)	Mean = 24 (7.7)		
(16%)						
**Group 3**	z = −0.4	z = −0.03	z = 0.2	z = 1.3	4.8 (2.7)	40 (7.8)
n = 36	Mean = 20 (7.9)	Mean = 7.5 (3.6)	Mean = 8.2 (2.9)	Mean = 62 (7.7)		
(27%)						
**Group 4**	z = −0.7	z = 0.3	z = −0.1	z = 0.1	6.0 (2.5)	40 (9.0)
n = 41	Mean = 16 (7.0)	Mean = 8.6 (3.3)	Mean = 7.5 (3.0)	Mean = 44 (5.7)		
(30%)						

z is a measure of distance from the mean with each point representing a standard deviation. Relatively younger adults with stronger athletic identity and middle-aged adults with lower athletic identity and greater feelings of distress regarding sensations tended to have greater pain intensity on average.

There were no differences in mean capability between statistical groups. Group 1 (younger adults with stronger athletic identity) and Group 4 (middle-aged adults with lower athletic identity and greater feelings of distress) had relatively greater pain intensity on average.

## Discussion

Stronger athletic identities are associated with higher levels of self-esteem and confidence, enhanced athletic performance, and better social relations [10]. And perceived or actual loss of a relatively strong athletic identity is associated with greater symptoms of depression during recovery from injury [10]. Evidence that levels of athletic identity mediate or moderate the relationship between thoughts and feelings about sensations and levels of discomfort and incapability has the potential to inform more effective strategies for athlete health and performance. Absence of mediation and moderation effects might suggest that athletic identities are not more impactful than other identities on the important relationship between mindsets and health. In a cross-sectional study of people seeking musculoskeletal specialty care from a sports unit, there was no relationship between athletic identity and levels of discomfort and incapability, precluding mediation and moderation analysis. An exploratory cluster analysis suggested potential relationships of groupings defined by age and athletic identity with symptom intensity.

### Limitations

This study has several limitations. First, we used a validated and commonly used measure of athletic identity, but it may be a difficult construct to quantify. If there is a sense that levels of athletic identity might be important to health, it could be worthwhile to test other measures of athletic identity or to explore development of new measures. Second, we enrolled patients at sports specialty offices, but approximately half of the participants identified as athletes. This could be a strength, as it allowed for a wide spectrum of levels of athletic identity. And most conditions experienced by athletes are also experienced by non-athletes. It also means that the results might be different among a group of high-level athletes. We designed the study to address moderation and mediation assuming there was a relationship between athletic identity and levels of discomfort and incapability. Our reasoning was that approaching the hypothesis with 2 statistical analyses could provide a more efficient and meaningful preliminary approach that could better help inform future studies. The exploratory, hypothesis-generating cluster analysis was then undertaken after the absence of an association rendered the planned mediation and moderation analysis infeasible.

Our priori size calculation was powered for the primary regression model. Moderation and mediation analyses generally require larger samples, and because there was no bivariate association between athletic identity and the outcomes, those analyses were not performed. We therefore cannot determine whether the study would have been adequately powered for them. We also did not construct a multivariable model combining athletic identity, distress, unhelpful thoughts, and age; these variables are related, raising concern for collinearity, and athletic identity was not associated with the outcomes in bivariate analyses. *Does athletic identity mediate or moderate the relationship between thoughts and feelings and level of capability and pain intensity?*

The finding that levels of athletic identity is not related to symptom intensity suggests that specific identities such as an athletic identity may not mediate or moderate the relationship between levels of comfort/capability and less healthy mindsets. This is consistent with a study of 360 adult UK and Irish athletes and 384 non-athletes surveyed after emergence from a COVID-19 lockdown that found a modest correlation between athletic identity and symptoms of anxiety and loneliness, no association with symptoms of depression, and no differences in resilience compared to non-athletes [11]. Another study identified diminished athletic identity over 2 years during recovery from anterior cruciate ligament reconstruction, more so among people who had a slower recovery trajectory, suggesting an evolution of athletic identity [12]. A meta-analysis of quantitative and qualitative studies of athletic identity found that formation of an athletic identity as an adolescent is associated with some health benefits but may also be associated with greater difficulty of adapatation of their identity when separating from sport due to injury, age, or loss of opportunity (e.g., graduation) [13]. In a study of former Division I college athletes and nonathletes at those schools, former athletes had greater limitations in daily activities, including fewer self-reported hours of both aerobic (1.9 vs. 4.3 hours for non-athletes) and anaerobic exercise (0.6 hours vs. 2 hours) [14]. When an athlete retires, identity evolution may be a part of maintaining a sense of meaning, purpose, and belonging. One can argue that this may be true for all identities. That might be an important aspect of good health: evolving one’s narrative identity to match one’s body as it ages and experiences disease or injury.

### Relationship of statistical groupings by mean age and mindset to athletic identity

The finding in an exploratory cluster analysis that a relatively younger grouping with stronger athletic identity tended to have higher mean pain intensity can be variably interpreted. Because athletic identity was not correlated with pain intensity in bivariate analysis and this grouping also differs in mean age, we regard this observation as hypothesis-generating rather than evidence of an effect on athletic identity. Any association of athletic identity with symptom intensity seems modest. The evidence that return to sport after ACL or shoulder instability surgery is associated with misinterpretation of painful activity as harmful (kinesiophobia) suggests that mindsets may be particularly relevant to recovery alongside musculoskeletal pathophysiology and athletic identity during recovery [15]. Our hypothesis is that perceived threat to athletic identity is no more or less associated with greater levels of discomfort and incapability than perceived threat to any other cherished social or occupational role, but our patient population and analysis were not able to confirm or refute this hypothesis.

## Conclusion

The finding of no direct association between athletic identity and pain intensity or capability alongside notable associations with less healthy thoughts and feelings--many of which address threats to cherished roles and identities--suggests that athletic identity, and perhaps other identities are less important than the perceived threat to one’s self-identity in general. The exploratory cluster analysis supports the hypothesis that distress, rather than athletic identity, could be a more important correlate of pain intensity. Many musculoskeletal pathophysiologies are permanent, senescent or post-traumatic pathophysiologies in particular. But an evolution in one’s narrative identity is always available. Adapting one’s identity to match pathophysiology and impairment--healthy thoughts and feelings regarding bodily sensations---is good for one’s health [16–19], perhaps independent of specific identities.
