# Supplementary material for: Is there a correlation between levels of incapability and athletic identity?
Source: PLoS One. 2026 Sep 30;21(9):e0359550. doi: 10.1371/journal.pone.0359550 (PMC13626326; doi:10.1371/journal.pone.0359550)
Supplement: Supplementary file 1 [file pone.0359550.s002.docx]

Dear Dr. Gunaydin,

Thank you for considering our manuscript titled “Is there a correlation between levels of incapability and athletic identity?” We revised the manuscript according to the guidance of the editor and reviewers as follows:

General Comments:

1. Please ensure that your manuscript meets PLOS ONE's style requirements, including those for file naming.

**Response:** OK

**Changes:** We reviewed the manuscript against the PLOS One Title/Author/Affiliations and Manuscript Body formatting guidelines. We adjusted the affiliations to be indicated by number, and the corresponding author is designated with an asterisk and listed by email only. Equal-contribution authors are marked with the pilcrow. References are cited numerically in order of first mention.

2. In the ethics statement in the Methods, you have specified that verbal consent was obtained. Please provide additional details regarding how this consent was documented and witnessed, and state whether this was approved by the IRB

**Response:** We have added the requested detail.

**Changes:** We revised the ethics statement in the Methods to specify how verbal consent was obtained, documented, and witnessed, and to state that the IRB approved the use of verbal consent. The statement now reads: “Verbal informed consent was obtained from each participant by a member of the research team prior to survey completion. The University of Texas IRB approved a verbal consent combined with completion of the questionnaires representing informed consent. Formal written consent was not required”

3. In the online submission form you indicate that your data is not available for proprietary reasons and have provided a contact point for accessing this data. Please note that your current contact point is a co-author on this manuscript. According to our Data Policy, the contact point must not be an author on the manuscript and must be an institutional contact, ideally not an individual. Please revise your data statement to a non-author institutional point of contact, such as a data access or ethics committee, and send this to us via return email. Please also include contact information for the third-party organization, and please include the full citation of where the data can be found.

**Response:** This was an error.

**Changes:** The complete dataset has been deidentified, containing no PHI, and deposited in a public repository, where it can be accessed by any interested researcher without restriction. We have updated the data availability statement accordingly and are sending the revised statement via email as requested. The revised statement reads: “The data underlying the results presented in this study are publicly available from the Texas Data Repository, University of Texas at Austin. The dataset has been fully deidentified and contains no PHI.”

4. Please include your full ethics statement in the ‘Methods’ section of your manuscript file. In your statement, please include the full name of the IRB or ethics committee who approved or waived your study, as well as whether or not you obtained informed written or verbal consent. If consent was waived for your study, please include this information in your statement as well.

**Response:** We have added the full ethics statement to the Methods.

**Changes:** We added a complete ethics statement to the Methods section that names the approving committee and states the consent process. It reads: “This study was reviewed and approved by the University of Texas at Austin Institutional Review Board. “Verbal informed consent was obtained from each participant by a member of the research team prior to survey completion. The University of Texas IRB approved a verbal consent combined with completion of the questionnaires as representing informed consent. Formal written consent was not required”

5. If the reviewer comments include a recommendation to cite specific previously published works, please review and evaluate these publications to determine whether they are relevant and should be cited. There is no requirement to cite these works unless the editor has indicated otherwise.

**Response:** Done. Viewed the editor and reviewer comments and found no recommendations to cite specific and previous published works.

**Changes:** None.

**Editor comments:**
1. The alignment between the study’s main hypothesis and the results needs to be strengthened.
The primary aim of the study is to examine the moderating or mediating role of athletic identity in the relationships between pain and function. However, no significant relationship was found between athletic identity and pain intensity or function. Therefore, whilst it is understandable that moderation/mediation analyses could not be conducted, it is recommended that comments regarding the potential effects of athletic identity be made with greater caution in the discussion and conclusions sections of the article.

**Response:** We agree.

**Changes:** We tempered our language regarding athletic identity throughout the Discussion and Conclusions. The statements regarding the potential effects of athletic identity have been revised to be more cautious. In the Conclusion, the sentence "The findings from the study suggest that athletic identity is associated with greater pain intensity and this merits additional investigation." has been replaced with: "The finding of no direct association between athletic identity and pain intensity or capability alongside notable associations with less healthy thoughts and feelings--many of which address threats to cherished roles and identities--suggests that athletic identity, and perhaps other identities are less important than the perceived threat to one’s self-identity in general."

In the Discussion, the sentence "In the cluster analysis, we found a greater athletic identity was associated with higher mean pain intensity independent of thoughts and feelings about sensations." has been replaced with: "An exploratory cluster analysis suggested potential relationships of groupings defined by age and athletic identity with symptom intensity. "

In the Discussion section, the word "exploratory" has been inserted before "observation" in "The observation that, among relatively younger patients, greater athletic identity was associated with greater pain intensity," so the cautious framing is consistent throughout.

2. It should be made clearer that the findings from the cluster analysis are of an exploratory nature.
Although the cluster analyses conducted following the rejection of the primary hypothesis are of interest, these analyses are aimed more at generating new hypotheses than at testing predefined ones. Therefore, it should be emphasized that the results are exploratory rather than confirmatory in nature.

**Response:** We agree

**Changes:** We revised the manuscript to describe the cluster analysis as exploratory and hypothesis-generating throughout.

In the Abstract, we revised “We planned mediation/moderation analysis. When that was not feasible we added and unplanned, exploratory cluster analysis of mindset factors." This is similarly described in the Statistical analysis section. We changed "Cluster analysis identified" to "An exploratory cluster analysis identified" in both the Abstract Results and the Results. We also added a sentence in the Discussion clarifying that the cluster analyses were exploratory, were undertaken after the primary hypothesis was infeasible.

3. Causal interpretations should be avoided.
The study has a cross-sectional design. Consequently, it is not possible to draw conclusions suggesting that distress or maladaptive thoughts cause pain and functional impairment. Reverse associations are equally plausible. It is recommended that statements in the discussion section that imply causality be reviewed.

**Response:** We agree.

**Changes:** We reviewed the Discussion and Conclusion and revised statements that could imply causality to reflect the cross-sectional design and the possibility of reverse associations. We replaced language suggesting that distress or unhelpful thoughts directly lead to greater pain and incapability.

4. The representativeness of the sample in terms of athletic identity should be discussed.
Only approximately half of the participants identify themselves as athletes. The high proportion of individuals who consulted the sports medicine clinic but were not athletes may have made it difficult to investigate the effects of athletic identity. This situation should be addressed in greater detail as a significant limitation of the study.

**Response:** Diversity of the sample is important, but there might be different findings in a sample of athletes, as we discuss in the discussion section.

**Changes:** We revised discussion of this in the limitations section, though this was already discussed.

5. The adequacy of the sample size for moderation analyses should be explained.
In the article, the sample size was calculated based on regression analysis. However, moderation and mediation analyses generally require larger samples. Therefore, it should be discussed whether the study possesses sufficient statistical power to evaluate these analyses.

**Response:** We checked and our statistician was not familiar with moderation analyses and did a power analysis for linear regression.

**Changes:** We clarified the statistical analysis section and added the following to the Statistics:

“Due to an oversight, however, this calculation was framed around multivariable linear regression rather than moderation analysis, leaving the study underpowered for the latter. As a check, assuming a medium effect size (Cohen’s f^2^ = 0.15), a two-sided significance level of 0.05, 80% statistical power, and eight predictor variables, the required sample size was estimated at 109 participants, which our sample met. However, because athletic identity was not associated with pain intensity or capability in bivariate analysis, the mediation/moderation analysis was not conducted, and this error had no bearing on our results or conclusions.”

6. Some statements in the Conclusions section do not appear to be fully consistent with the findings.
Whilst various sections of the article state that there is no association between athletic identity and pain or function, the Conclusions section contains statements suggesting that athletic identity is associated with higher pain intensity. It is recommended that these statements be made more consistent.

**Response:** We agree.

**Changes:** We revised the Conclusion to be consistent with the finding of no association between athletic identity and pain intensity or capability. We replaced the final sentence with text clarifying that we did not find a direct association between athletic identity and pain or capability. This makes clear that distress, rather than athletic identity, was the consistent correlate. These revisions aligned with responses to comments 1 and 2.

7. The names given to the cluster groups could be made more neutral.
Descriptions such as ‘Young and hopeful’ and ‘distressed non-athletes’ may give the reader the impression of subjective interpretation. It would be appropriate to present the groups using more objective and descriptive terms.

**Response:** Labels assist with understanding, but we can try to be more descriptive than interpretive.

**Changes:** The revised descriptors are: Group 1: younger adults with stronger athletic identity, Group 2: younger adults with lower feelings of distress, Group 3: older adults with lower athletic identity and somewhat greater unhelpful thoughts, and Group 4: middle-aged adults with lower athletic identity and greater feelings of distress.

8. The handling of missing data should be explained in greater detail.
In particular, there is a high proportion (30%) of missing data in the income variable. It should be specified how these missing data points were handled in the analyses.

**Response:** Prefer not to answer was an available choice and was treated as a specific category in the analysis.

**Changes:** We describe this in the revised manuscript.

9. The inclusion of multivariate analyses could strengthen the study.
Evaluating variables such as athletic identity, distress, unhelpful thoughts and age within the same model could help to clarify their independent effects more clearly.

**Response:** We have added our rationale for not performing a multivariable analysis.

**Changes:** We added context to the Statistical analysis section and the limitations. We note that the variables are related, raising concern for collinearity, and that athletic identity had no bivariate association with either pain intensity or capability, so it could not contribute meaningfully to multivariable outcomes**.**
