# Supplementary material for: Is there a correlation between levels of incapability and athletic identity?
Source: PLoS One. 2026 Sep 30;21(9):e0359550. doi: 10.1371/journal.pone.0359550 (PMC13626326; doi:10.1371/journal.pone.0359550)
Supplement: Peer review history

# Peer review: Decision Letter 0

**DOI:** 10.1371/journal.pone.0359550.r001

**Authors:** Gurkan Gunaydin

8 Jun 2026

PONE-D-25-51613

Is there a correlation between levels of incapability and athletic identity?

PLOS One?

Dear Dr. Ring,

Thank you for submitting your manuscript to PLOS ONE. After careful consideration, we feel that it has merit but does not fully meet PLOS ONE’s publication criteria as it currently stands. Therefore, we invite you to submit a revised version of the manuscript that addresses the points raised during the review process.

Please submit your revised manuscript by Jul 23 2026 11:59PM. If you will need more time than this to complete your revisions, please reply to this message or contact the journal office at plosone@plos.org. When you're ready to submit your revision, log on to https://www.editorialmanager.com/pone/ and select the 'Submissions Needing Revision' folder to locate your manuscript file.

- A letter that responds to each point raised by the academic editor and reviewer(s). You should upload this letter as a separate file labeled 'Response to Reviewers'.
- A marked-up copy of your manuscript that highlights changes made to the original version. You should upload this as a separate file labeled 'Revised Manuscript with Track Changes'.
- An unmarked version of your revised paper without tracked changes. You should upload this as a separate file labeled 'Manuscript'.

If you would like to make changes to your financial disclosure, please include your updated statement in your cover letter. Guidelines for resubmitting your figure files are available below the reviewer comments at the end of this letter.

If applicable, we recommend that you deposit your laboratory protocols in protocols.io to enhance the reproducibility of your results. Protocols.io assigns your protocol its own identifier (DOI) so that it can be cited independently in the future. For instructions see: https://journals.plos.org/plosone/s/submission-guidelines#loc-laboratory-protocols. Additionally, PLOS ONE offers an option for publishing peer-reviewed Lab Protocol articles, which describe protocols hosted on protocols.io. Read more information on sharing protocols at https://plos.org/protocols?utm_medium=editorial-email&utm_source=authorletters&utm_campaign=protocols.

As the corresponding author, your ORCID iD is verified in the submission system and will appear in the published article. PLOS supports the use of ORCID, and we encourage all coauthors to register for an ORCID iD and use it as well. Please encourage your coauthors to verify their ORCID iD within the submission system before final acceptance, as unverified ORCID iDs will not appear in the published article. *Only*  the individual author can complete the verification step; PLOS staff *cannot*  verify ORCID iDs on behalf of authors.

We look forward to receiving your revised manuscript.

Kind regards,

Gurkan Gunaydin

Academic Editor

PLOS One

Journal Requirements:

When submitting your revision, we need you to address these additional requirements.

1. Please ensure that your manuscript meets PLOS ONE's style requirements, including those for file naming. The PLOS ONE style templates can be found at

https://journals.plos.org/plosone/s/file?id=wjVg/PLOSOne_formatting_sample_main_body.pdf and

https://journals.plos.org/plosone/s/file?id=ba62/PLOSOne_formatting_sample_title_authors_affiliations.pdf

2. In the ethics statement in the Methods, you have specified that verbal consent was obtained. Please provide additional details regarding how this consent was documented and witnessed, and state whether this was approved by the IRB

3. In the online submission form you indicate that your data is not available for proprietary reasons and have provided a contact point for accessing this data. Please note that your current contact point is a co-author on this manuscript. According to our Data Policy, the contact point must not be an author on the manuscript and must be an institutional contact, ideally not an individual. Please revise your data statement to a non-author institutional point of contact, such as a data access or ethics committee, and send this to us via return email. Please also include contact information for the third party organization, and please include the full citation of where the data can be found.

4. Please include your full ethics statement in the ‘Methods’ section of your manuscript file. In your statement, please include the full name of the IRB or ethics committee who approved or waived your study, as well as whether or not you obtained informed written or verbal consent. If consent was waived for your study, please include this information in your statement as well.

5. If the reviewer comments include a recommendation to cite specific previously published works, please review and evaluate these publications to determine whether they are relevant and should be cited. There is no requirement to cite these works unless the editor has indicated otherwise.

Additional Editor Comments:

Dear Authors,

Please find below the recommendations for the revision of your manuscript. We are well aware that the evaluation process of your manuscript has taken a considerable amount of time; however, I would like to emphasize that your work has been handled with utmost care and diligence at every stage.

1. The alignment between the study’s main hypothesis and the results needs to be strengthened.

The primary aim of the study is to examine the moderating or mediating role of athletic identity in the relationships between pain and function. However, no significant relationship was found between athletic identity and pain intensity or function. Therefore, whilst it is understandable that moderation/mediation analyses could not be conducted, it is recommended that comments regarding the potential effects of athletic identity be made with greater caution in the discussion and conclusions sections of the article.

2. It should be made clearer that the findings from the cluster analysis are of an exploratory nature.

Although the cluster analyses conducted following the rejection of the primary hypothesis are of interest, these analyses are aimed more at generating new hypotheses than at testing predefined ones. Therefore, it should be emphasised that the results are exploratory rather than confirmatory in nature.

3. Causal interpretations should be avoided.

The study has a cross-sectional design. Consequently, it is not possible to draw conclusions suggesting that distress or maladaptive thoughts cause pain and functional impairment. Reverse associations are equally plausible. It is recommended that statements in the discussion section that imply causality be reviewed.

4. The representativeness of the sample in terms of athletic identity should be discussed.

Only approximately half of the participants identify themselves as athletes. The high proportion of individuals who consulted the sports medicine clinic but were not athletes may have made it difficult to investigate the effects of athletic identity. This situation should be addressed in greater detail as a significant limitation of the study.

5. The adequacy of the sample size for moderation analyses should be explained.

In the article, the sample size was calculated based on regression analysis. However, moderation and mediation analyses generally require larger samples. Therefore, it should be discussed whether the study possesses sufficient statistical power to evaluate these analyses.

6. Some statements in the Conclusions section do not appear to be fully consistent with the findings.

Whilst various sections of the article state that there is no association between athletic identity and pain or function, the Conclusions section contains statements suggesting that athletic identity is associated with higher pain intensity. It is recommended that these statements be made more consistent.

7. The names given to the cluster groups could be made more neutral.

Descriptions such as ‘Young and hopeful’ and ‘distressed non-athletes’ may give the reader the impression of subjective interpretation. It would be appropriate to present the groups using more objective and descriptive terms.

8. The handling of missing data should be explained in greater detail.

In particular, there is a high proportion (30%) of missing data in the income variable. It should be specified how these missing data points were handled in the analyses.

9. The inclusion of multivariate analyses could strengthen the study.

Evaluating variables such as athletic identity, distress, unhelpful thoughts and age within the same model could help to clarify their independent effects more clearly.

[Note: HTML markup is below. Please do not edit.]

Reviewers' comments:

Reviewer's Responses to Questions

**Comments to the Author**

1. Is the manuscript technically sound, and do the data support the conclusions?

Reviewer #1: Yes

**********

2. Has the statistical analysis been performed appropriately and rigorously?

Reviewer #1: Yes

**********

3. Have the authors made all data underlying the findings in their manuscript fully available?

The PLOS Data policy

Reviewer #1: Yes

**********

4. Is the manuscript presented in an intelligible fashion and written in standard English?

Reviewer #1: Yes

**********

Reviewer #1: A most interesting work. The way patients perceive the world, and themselves has always had an influence on pain and functional capability. I find it interesting that as always, negative feelings seem to be correlated much more strongly than confidence or positive feelings. This is in lign with all current literature regarding the subject.

**********

what does this mean?). If published, this will include your full peer review and any attached files.

If you choose “no”, your identity will remain anonymous but your review may still be made public.

**Do you want your identity to be public for this peer review?**  For information about this choice, including consent withdrawal, please see our Privacy Policy

Reviewer #1: No

**********

[NOTE: If reviewer comments were submitted as an attachment file, they will be attached to this email and accessible via the submission site. Please log into your account, locate the manuscript record, and check for the action link "View Attachments". If this link does not appear, there are no attachment files.]

To ensure your figures meet our technical requirements, please review our figure guidelines: https://journals.plos.org/plosone/s/figures

You may also use PLOS’s free figure tool, NAAS, to help you prepare publication quality figures: https://journals.plos.org/plosone/s/figures#loc-tools-for-figure-preparation.

NAAS will assess whether your figures meet our technical requirements by comparing each figure against our figure specifications.

# Author comment: Author response to Decision Letter 1

**DOI:** 10.1371/journal.pone.0359550.r002

9 Sep 2026

Thank you for taking the time to review this research and proving feedback to improve it. For your convenience, we have also attached our responses to each comment below.

1. The alignment between the study’s main hypothesis and the results needs to be strengthened.

The primary aim of the study is to examine the moderating or mediating role of athletic identity in the relationships between pain and function. However, no significant relationship was found between athletic identity and pain intensity or function. Therefore, whilst it is understandable that moderation/mediation analyses could not be conducted, it is recommended that comments regarding the potential effects of athletic identity be made with greater caution in the discussion and conclusions sections of the article.

Response: We agree.

Changes: We tempered our language regarding athletic identity throughout the Discussion and Conclusions. The statements regarding the potential effects of athletic identity have been revised to be more cautious. In the Conclusion, the sentence "The findings from the study suggest that athletic identity is associated with greater pain intensity and this merits additional investigation." has been replaced with: "The finding of no direct association between athletic identity and pain intensity or capability alongside notable associations with less healthy thoughts and feelings--many of which address threats to cherished roles and identities--suggests that athletic identity, and perhaps other identities are less important than the perceived threat to one’s self-identity in general."

In the Discussion, the sentence "In the cluster analysis, we found a greater athletic identity was associated with higher mean pain intensity independent of thoughts and feelings about sensations." has been replaced with: "An exploratory cluster analysis suggested potential relationships of groupings defined by age and athletic identity with symptom intensity. "

In the Discussion section, the word "exploratory" has been inserted before "observation" in "The observation that, among relatively younger patients, greater athletic identity was associated with greater pain intensity," so the cautious framing is consistent throughout.

2. It should be made clearer that the findings from the cluster analysis are of an exploratory nature.

Although the cluster analyses conducted following the rejection of the primary hypothesis are of interest, these analyses are aimed more at generating new hypotheses than at testing predefined ones. Therefore, it should be emphasized that the results are exploratory rather than confirmatory in nature.

Response: We agree

Changes: We revised the manuscript to describe the cluster analysis as exploratory and hypothesis-generating throughout.

In the Abstract, we revised “We planned mediation/moderation analysis. When that was not feasible we added and unplanned, exploratory cluster analysis of mindset factors." This is similarly described in the Statistical analysis section. We changed "Cluster analysis identified" to "An exploratory cluster analysis identified" in both the Abstract Results and the Results. We also added a sentence in the Discussion clarifying that the cluster analyses were exploratory, were undertaken after the primary hypothesis was infeasible.

3. Causal interpretations should be avoided.

The study has a cross-sectional design. Consequently, it is not possible to draw conclusions suggesting that distress or maladaptive thoughts cause pain and functional impairment. Reverse associations are equally plausible. It is recommended that statements in the discussion section that imply causality be reviewed.

Response: We agree.

Changes: We reviewed the Discussion and Conclusion and revised statements that could imply causality to reflect the cross-sectional design and the possibility of reverse associations. We replaced language suggesting that distress or unhelpful thoughts directly lead to greater pain and incapability.

4. The representativeness of the sample in terms of athletic identity should be discussed.

Only approximately half of the participants identify themselves as athletes. The high proportion of individuals who consulted the sports medicine clinic but were not athletes may have made it difficult to investigate the effects of athletic identity. This situation should be addressed in greater detail as a significant limitation of the study.

Response: Diversity of the sample is important, but there might be different findings in a sample of athletes, as we discuss in the discussion section.

Changes: We revised discussion of this in the limitations section, though this was already discussed.

5. The adequacy of the sample size for moderation analyses should be explained.

In the article, the sample size was calculated based on regression analysis. However, moderation and mediation analyses generally require larger samples. Therefore, it should be discussed whether the study possesses sufficient statistical power to evaluate these analyses.

Response: We checked and our statistician was not familiar with moderation analyses and did a power analysis for linear regression.

Changes: We clarified the statistical analysis section and added the following to the Statistics:

“Due to an oversight, however, this calculation was framed around multivariable linear regression rather than moderation analysis, leaving the study underpowered for the latter. As a check, assuming a medium effect size (Cohen’s f2 = 0.15), a two-sided significance level of 0.05, 80% statistical power, and eight predictor variables, the required sample size was estimated at 109 participants, which our sample met. However, because athletic identity was not associated with pain intensity or capability in bivariate analysis, the mediation/moderation analysis was not conducted, and this error had no bearing on our results or conclusions.”

6. Some statements in the Conclusions section do not appear to be fully consistent with the findings.

Whilst various sections of the article state that there is no association between athletic identity and pain or function, the Conclusions section contains statements suggesting that athletic identity is associated with higher pain intensity. It is recommended that these statements be made more consistent.

Response: We agree.

Changes: We revised the Conclusion to be consistent with the finding of no association between athletic identity and pain intensity or capability. We replaced the final sentence with text clarifying that we did not find a direct association between athletic identity and pain or capability. This makes clear that distress, rather than athletic identity, was the consistent correlate. These revisions aligned with responses to comments 1 and 2.

7. The names given to the cluster groups could be made more neutral.

Descriptions such as ‘Young and hopeful’ and ‘distressed non-athletes’ may give the reader the impression of subjective interpretation. It would be appropriate to present the groups using more objective and descriptive terms.

Response: Labels assist with understanding, but we can try to be more descriptive than interpretive.

Changes: The revised descriptors are: Group 1: younger adults with stronger athletic identity, Group 2: younger adults with lower feelings of distress, Group 3: older adults with lower athletic identity and somewhat greater unhelpful thoughts, and Group 4: middle-aged adults with lower athletic identity and greater feelings of distress.

8. The handling of missing data should be explained in greater detail.

In particular, there is a high proportion (30%) of missing data in the income variable. It should be specified how these missing data points were handled in the analyses.

Response: Prefer not to answer was an available choice and was treated as a specific category in the analysis.

Changes: We describe this in the revised manuscript.

9. The inclusion of multivariate analyses could strengthen the study.

Evaluating variables such as athletic identity, distress, unhelpful thoughts and age within the same model could help to clarify their independent effects more clearly.

Response: We have added our rationale for not performing a multivariable analysis.

Changes: We added context to the Statistical analysis section and the limitations. We note that the variables are related, raising concern for collinearity, and that athletic identity had no bivariate association with either pain intensity or capability, so it could not contribute meaningfully to multivariable outcomes.

Attachment Submitted filename: Response to reviewers.docx

# Editor report: Decision Letter 1

**DOI:** 10.1371/journal.pone.0359550.r003

**Authors:** Gurkan Gunaydin

15 Sep 2026

Is there a correlation between levels of incapability and athletic identity?

PONE-D-25-51613R1

Dear Dr. Ring,

We’re pleased to inform you that your manuscript has been judged scientifically suitable for publication and will be formally accepted for publication once it meets all outstanding technical requirements.

Within one week, you’ll receive an e-mail detailing the required amendments. When these have been addressed, you’ll receive a formal acceptance letter and your manuscript will be scheduled for publication.

An invoice will be generated when your article is formally accepted. Please note, if your institution has a publishing partnership with PLOS and your article meets the relevant criteria, all or part of your publication costs will be covered. Please make sure your user information is up-to-date by logging into Editorial Manager at Editorial Manager® and clicking the ‘Update My Information' link at the top of the page. For questions related to billing, please contact billing support.

If your institution or institutions have a press office, please notify them about your upcoming paper to help maximize its impact. If they’ll be preparing press materials, please inform our press team as soon as possible -- no later than 48 hours after receiving the formal acceptance. Your manuscript will remain under strict press embargo until 2 pm Eastern Time on the date of publication. For more information, please contact onepress@plos.org.

Kind regards,

Gurkan Gunaydin

Academic Editor

PLOS One

Additional Editor Comments (optional):

Dear Authors,

The corrections made appear to be sufficient.

Reviewers' comments:

# Editor report: Acceptance letter

**DOI:** 10.1371/journal.pone.0359550.r004

**Authors:** Gurkan Gunaydin

PONE-D-25-51613R1

PLOS One

Dear Dr. Ring,

I'm pleased to inform you that your manuscript has been deemed suitable for publication in PLOS One. Congratulations! Your manuscript is now being handed over to our production team.

At this stage, our production department will prepare your paper for publication. This includes ensuring the following:

* All references, tables, and figures are properly cited

* All relevant supporting information is included in the manuscript submission,

* There are no issues that prevent the paper from being properly typeset

You will receive further instructions from the production team, including instructions on how to review your proof when it is ready. Please keep in mind that we are working through a large volume of accepted articles, so please give us a few days to review your paper and let you know the next and final steps.

Lastly, if your institution or institutions have a press office, please let them know about your upcoming paper now to help maximize its impact. If they'll be preparing press materials, please inform our press team within the next 48 hours. Your manuscript will remain under strict press embargo until 2 pm Eastern Time on the date of publication. For more information, please contact onepress@plos.org.

You will receive an invoice from PLOS for your publication fee after your manuscript has reached the completed accept phase. If you receive an email requesting payment before acceptance or for any other service, this may be a phishing scheme. Learn how to identify phishing emails and protect your accounts at https://explore.plos.org/phishing.

If we can help with anything else, please email us at customercare@plos.org.

Thank you for submitting your work to PLOS One and supporting open access.

Kind regards,

PLOS One Editorial Office Staff

on behalf of

Assoc. Prof. Gurkan Gunaydin

Academic Editor

PLOS One
